# Comparative Evaluation of Wild Plum (*Prunus spinosa* L.) Stone Fruits and Leaves: Insights into Nutritional Composition, Antioxidant Properties, and Polyphenol Profile

**DOI:** 10.3390/foods15010142

**Published:** 2026-01-02

**Authors:** Petru Alexandru Vlaicu, Iulia Varzaru, Raluca Paula Turcu, Alexandra Gabriela Oancea, Arabela Elena Untea

**Affiliations:** Feed and Food Quality Department, National Research and Development Institute for Animal Biology and Nutrition, Balotesti, 077015 Ilfov, Romania; alexandru.vlaicu@outlook.com (P.A.V.); iulia.maros@ibna.ro (I.V.); raluca.turcu@ibna.ro (R.P.T.); alexandra.oancea@ibna.ro (A.G.O.)

**Keywords:** antioxidants, bioactive compounds, nutrients, *Prunus spinosa*, wild flora, polyphenols, fruits, leaves

## Abstract

*Prunus spinosa* L. is a shrub with nutritional potential, yet limited information is available on the composition of its stone fruit and leaves. This study aimed to investigate and compare the macro and micronutrients, fatty acid profile, and lipophilic and hydrophilic antioxidant compounds in fruits and leaves, as well as their potential functional properties. The results revealed that leaves contain higher crude protein (10.94%) than fruits (6.46%) but lower crude fiber (13.86% compared to 22.16%). The iron (370.37 mg/kg) and manganese (43.57 mg/kg) were significantly higher (*p* < 0.05) in leaves than in fruits (44.87 versus 7.02 mg/kg). The fruits’ lipid profile was rich in monounsaturated fatty acids (56.8%), whereas the leaves showed higher saturated fatty acids (38.3%) and polyunsaturated fatty acids (43.7%). The leaves also presented significantly higher n-3 content (25.2%) compared with fruits (1.80%). The antioxidant compounds were higher in the leaves, with total vitamin E exceeding 1268 mg/kg, primarily α-tocopherol (1214.98 mg/kg) isoform, lutein (409.38 mg/kg), and astaxanthin (3.74 mg/kg), compared with only 74.75 mg/kg total vitamin E in the fruits. The total hydroxycinnamic polyphenols in leaves were 92.63% higher in leaves than in fruits, with anthocyanins at 63.23% and flavonols at 95.82%. Although the leaves demonstrated superior antioxidant potential and mineral content compared to the fruits, making it a promising candidate for nutraceutical and functional food applications, the fruits maintained a healthier lipid profile suitable for dietary fat intake. This comparative analysis highlights the distinct nutritional and bioactive composition of *Prunus spinosa* co-products.

## 1. Introduction

Numerous wild plant species have long contributed to human diets as sources of essential nutrients and bioactive compounds with beneficial effects. In recent years, interest in underutilized or lesser-known fruits and leafy tissues has increased, mostly due to their potential usage and role in sustainable nutrition, natural antioxidant supply, and valorization of non-commercial plant resources. Among these underutilized plants, *Prunus spinosa* L. (wild plum) is a widespread wild shrub of the *Rosaceae* family native to Europe and western Asia. Stone fruits are known for their tart and astringent flavor but are mostly used for medicinal purposes and food preparations [1], especially in liqueurs (such as pacharán). They have attracted interest due to their phytochemical profile and potential health benefits [2].

Studies on *Prunus spinosa* fruits highlight their density of health-promoting compounds; they are rich in carbohydrates, with a low-fat content and considerable mineral content (potassium, calcium, and magnesium) [3]. Other trace elements in fruits (iron, zinc, and manganese) have also been reported, suggesting that the fruits could contribute to micronutrient intake in human diets [4]. However, in leaves the mineral composition has received little attention [5], suggesting the need of additional comparative studies among the different plant parts. The literature data showed that the compositional profile of *Prunus spinosa* stone fruits presents a complex array of bioactive compounds. These include phenolic acids, flavonoids (such as rutin, quercetin, and kaempferol), and anthocyanins (including cyanidin and peonidin glycosides) [1]. Previous comparative studies on the fruits of *Prunus spinosa* consistently exhibited higher phenolic concentration and antioxidant capacity than cultivated plum species, such as *Prunus domestica* and *Prunus cerasifera* [6,7,8]. Lipophilic compounds are critical antioxidants in both plant physiology and human health. Recently [5] reported that *Prunus spinosa* fruits contain 97 mg/kg of α-tocopherol, with additional γ- and δ-tocopherols isoforms determined. The same study showed that lutein and zeaxanthin reach 123 mg/kg, levels comparable to or exceeding those of some cultivated berries [1,9,10]. However, the literature data are scarce in reporting these antioxidants in leaves, despite the fact that they often contain higher carotenoid contents and bioactive compounds than fruits [11,12,13,14]. Although *Prunus spinosa* fruits are scarce in lipids, recent analyses reported linoleic acid (C18:2) as the dominant fatty acid (57 to 64%), followed by oleic acid (18 to 29%) in oil kernels [15]. However, data regarding the fatty acid composition of *Prunus spinosa* fruits and leaves is scarce, revealing a gap in understanding its full nutritional potential. When compared with other wild fruits, such as hawthorn (*Crataegus* spp.) [16], black chokeberry (*Aronia melanocarpa* L.) [11], blackberry (*Rubus fructicosus*) [13,14], or blackcurrant (*Ribes nigrum* L.) [12], *Prunus spinosa* exhibits stronger antioxidant capacity [2]. However, most previous studies have focused exclusively on either fruits or their by-products, without considering the potential of leaves. Nevertheless, considering the need for sustainable alternatives for both human and animal nutrition, in recent years, more preliminary studies have highlighted the nutritional potential and phytochemical analysis of different leaves compared to fruits [11,12,13,17] for potential utilization and exploitation. In this light, detailed evaluation of both plant parts is needed to determine their nutritional and phytochemical contributions, and to explore opportunities for their inclusion in functional foods, nutraceuticals, and animal nutrition.

In this context, the present study aims to provide a comparative evaluation of *Prunus spinosa* stone fruits and leaves, focusing on their nutritional, mineral, lipid composition, antioxidant capacity, and phenolic content, as well as their potential usage. This study would contribute to a deeper understanding of the health-promoting potential of wild *Prunus spinosa* co-products harvested in Romania and supports its potential application in the development of functional foods, nutraceuticals, and other industries.

## 2. Materials and Methods

### 2.1. Plant Material

**Figure 1:**
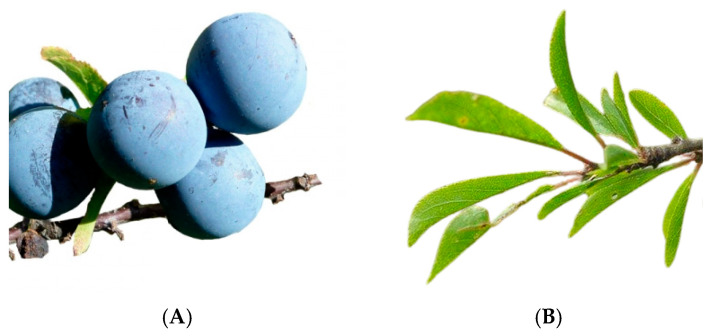
Presentation of *Prunus spinosa* L. (**A**) fruits; (**B**) leaves.

The leaves of wild plum shrub (*Prunus spinosa* L.) were collected in mid-August and the fruits in late-September when they were ready for ripening from wild flora located near Iasi County (47.1667° N, 27.4167° E), Romania. The plant material (fruits and leaves) was dried in an oven at 65 °C for 24 to 48 h under continuous air circulation until constant weight was achieved (Figure 1A,B). The duration selected ensured sufficient moisture removal from leaves in 24 h and from fruits in 48 h while minimizing degradation of heat-sensitive biologically active compounds. After, the plant material was ground into a fine powder using a Grindomix GM 200 mill (Retsch, Haan, Germany) with a 0.5 mm sieve and stored in paper bags until further laboratory analyses were conducted.

### 2.2. Determination of Macro and Micronutrient Content

#### 2.2.1. Macronutrients

The primary chemical composition analysis of the fruits and leaves was carried out following the authorized methods and techniques recommended by the Association of Official Analytical Chemists [18]. Crude protein (CP) was determined by the Kjeldahl method (Kjeltec auto 1030 Tecator Instruments, Höganäs, Sweden). The crude fat (EE) was determined using a Soxhlet apparatus by extraction in organic solvents (Soxtec 2055 Foss Tecator, Höganäs, Sweden). The crude fiber (CF) was determined by the method with intermediary filtration (Fibertec 2010 System Foss Tecator, Höganäs, Sweden).

#### 2.2.2. Micronutrients

The content of copper (Cu), iron (Fe), manganese (Mn), and zinc (Zn) in the fruits and leaves of *Prunus spinosa* samples was determined by flame atomic absorption spectrometry (FAAS, Thermo Electron SOLAAR M6 Dual Zeeman Comfort, Cambridge, UK) after microwave digestion (Berghof, Eningen, Germany) as described by [19].

### 2.3. Determination of Fatty Acids Composition

The fatty acid composition was determined using a gas chromatograph Perkin-Elmer Clarus 500 (Waltham, MA, USA) equipped with a flame ionization detector and capillary separation column with a high polar stationary phase TRACE TR-Fame (Thermo Electron, Waltham, MA, USA), with dimensions of 60 m × 0.25 mm × 0.25 μm. The extracted fat was mixed with 50 mL of acidified methanol and boiled under reflux with a water bath (FALC WB-U6, FALC Instruments, Treviglio, Italy) for 25–30 min. After cooling, it was mixed with distilled water and hexane, stirred, and transferred to a separation funnel. The organic layer was rinsed with distilled water, dried using anhydrous Na_2_SO_4_, and concentrated using a rotary evaporator [20]. The amount of each fatty acid was expressed as g/100 g fatty acid methyl esters (FAME) after triplicate analyses and readings.

### 2.4. Extraction of Hydrosoluble and Liposoluble Antioxidant Compounds

The extraction of hydrosoluble antioxidants from leaves and fruits, for determination of total phenolic compounds (TPC) and antioxidant activity (DPPH), was performed using 80% methanol, according to the method previously described [21]. The samples were placed on a rotary shaker in the dark for 24 h, then centrifuged at 1500× *g* for 10 min using a refrigerated centrifuge (Sigma 3–16 KL, Laborzentrifugen GmbH, Osterode am Harz, Germany). The supernatant obtained was used for analysis.

The extraction of liposoluble antioxidants from leaves and fruits followed the procedure previously described [13]. Prior to extraction, a saponification step was necessary, involving the hydrolysis of samples with an ethanolic potassium hydroxide solution in a water bath for 30 min at 80 °C, followed by petroleum ether extraction.

#### 2.4.1. Determination of Total Phenolic Compounds (TPC)

The TPC was determined with the Folin–Ciocalteu reagent, as described previously [21]. Absorbance was measured at 732 nm using a Varioskan Lux microplate reader (Thermo Fisher Scientific, Waltham, MA, USA). The calculation of concentrations was performed based on a calibration curve (R^2^ = 0.9993), and the results were expressed as milligrams gallic acid equivalents per gram of dry matter (mg GAE/g dw).

#### 2.4.2. Determination of Antioxidant Activity by the DPPH Method

The DPPH radical scavenging activity was determined by the modified spectrophotometric method [22]. The decrease in absorbance at 517 nm was measured using the same Varioskan Lux microplate reader (Thermo Fisher Scientific, Waltham, MA, USA). The calibration curve was constructed using various concentrations of Trolox (6-hydroxy-2,5,7,8-tetramethylchroman-2-carboxylic acid), and the antioxidant activity of the samples was expressed as millimoles Trolox equivalents per kilogram of sample (mmol TE/kg).

#### 2.4.3. Determination of Xanthophylls Content

The xanthophyll content (lutein, zeaxanthin, astaxanthin) was determined using a high-performance liquid chromatography (HPLC) method, with a Perkin Elmer 200 series System (Shelton, CT, USA) and a UV detector (λ = 450 nm). The mobile phase consisted of acetone (75%), methanol (15%), and water (10%), and the separation was performed with a C18 reversed-phase column (Nucleodur, Macherey-Nagel, Germany), with dimensions: 5 µm, 250 mm × 4.60 mm interior diameter [13].

#### 2.4.4. Determination of Tocopherols Content

The determination of the vitamin E isomers was assessed using the HPLC method, with a Vanquish Core HPLC System (Thermo-Electron Corporation, Waltham, MA, USA), a PDA–UV detector (λ = 292 nm), and an Accucore C18 column (Thermo-Electron Corporation, Waltham, MA, USA), with the following dimensions: 4 µm particle size, 150 mm × 4.6 mm. The mobile phase consisted of methanol (96%) and water (4%).

#### 2.4.5. Polyphenols Profile Quantification

The profile of polyphenols was assessed using a liquid chromatographic method [23], a Vanquish Core HPLC system equipped with a DAD manufactured by Thermo Fisher Scientific (Bremen, Germany), and a BDS HyperSil C18 column (250 × 4 mm, 5 µm particle size) from Thermo Fisher Scientific (Bremen, Germany). The chromatographic method involves a binary gradient comprising acetic acid (1%) in distilled water (*v*/*v*) as solvent A, methanol as solvent B, and acetonitrile as solvent C, with a flow rate of 0.5 mL/min and an elution program as follows: 0–15 min: 5% solvent B, 5% solvent C; 15–20 min: 4% solvent B, 15% solvent C; 20–25 min: 3% solvent B, 25% solvent C; 25–40 min: 2% solvent B, 38% solvent C; 40–50 min: 5% solvent B, 5% solvent C. The standards of individual polyphenols were purchased from Sigma-Aldrich (Steinheim, Germany) and used for the identification and quantification of polyphenolic compounds. The results are expressed as mg/g.

### 2.5. Statistical Analyses

Statistical analysis was performed by one-way ANOVA using GraphPad Prism statistical software version 9.03, followed by Tukey’s post hoc test. Graphs were created using the same software, GraphPad Prism software version 9.03 (San Diego, CA, USA).

## 3. Results

### 3.1. Macronutrient Composition of Prunus Spinosa Stone Fruits and Leaves

**Figure 2:**
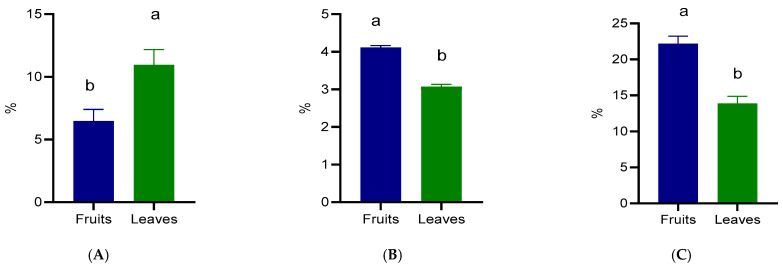
Crude protein (**A**), crude fat (**B**), and crude fiber (**C**) composition of wild *Prunus spinosa* stone fruits and leaves. Different letters (a,b) show significant differences at *p* < 0.05 according to ANOVA test; n = 3 represents 3 independent analyses, each performed with 3 analytical determinations.

The proximate composition of the fruits and leaves is presented in Figure 2. Considerable differences were observed between the two plant parts. The protein content was significantly higher (*p* < 0.05) in the leaves (10.94%) compared with the fruits (6.46%) (Figure 2A). On the other hand, the fat content was significantly higher (*p* <0.05) in the fruits (4.11%) than in the leaves (3.07%) (Figure 2B). The fiber content also followed an opposite trend to protein, with fruits containing 22.16% and leaves 13.86% fiber (Figure 2C).

### 3.2. Micronutrient Composition of Wild Prunus spinosa Stone Fruits and Leaves

The mineral composition is summarized in Table 1. Significant differences were observed between both plant parts for the analyzed micronutrients (*p* < 0.05), except for copper concentrations, which were similar in both fruits and leaves (*p* = 0.0951). The most significant differences (*p* < 0.001) were observed in manganese and iron, which were 83.88% and 87.88% higher, respectively, in leaves compared with fruits. The zinc content was also higher in leaves than in fruits.

### 3.3. Lipid Profile of Wild Prunus Spinosa Stone Fruits and Leaves

The lipid composition presented in Table 2 showed significant differences (*p* < 0.05) among individual fatty acids. The fruits were dominated by unsaturated lipids (UFA), especially oleic and linoleic content. In contrast, leaves were noted to contain significantly higher (*p* < 0.05) saturated fats (SFA), (palmitic and stearic), and α-linolenic acid. The fruits showed significantly higher (*p* < 0.05) UFA, lower SFA/UFA, and PUFA/MUFA, and an unbalanced n-6/n-3 ratio. In contrast, the leaves presented lower UFA and higher SFA/UFA, with moderate PUFA/MUFA ratio and a favorable n-6/n-3.

### 3.4. Total Polyphenols and DPPH Activity of Wild Prunus Spinosa Stone Fruits and Leaves

**Figure 3:**
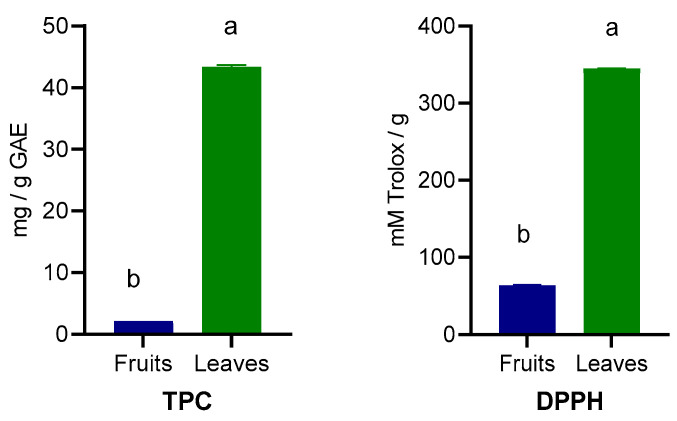
Total polyphenols content (TPC) and antioxidant capacity (DPPH) of wild *Prunus spinosa* stone fruits and leaves. Different letters (a,b) show significant differences at *p* < 0.05 according to ANOVA test; n = 3 represents 3 independent analyses, each performed with 3 analytical determinations.

The TPC and antioxidant capacity determined by DPPH activity are presented in Figure 3. The leaves contained significantly higher (*p* < 0.001) TPC (8.63 versus 173.44 mg/g GAE) content as well as higher DPPH activity (256.88 versus 1380.19 mM Trolox/g).

### 3.5. Xanthophyll Profile of Wild Prunus Spinosa Stone Fruits and Leaves

**Figure 4:**
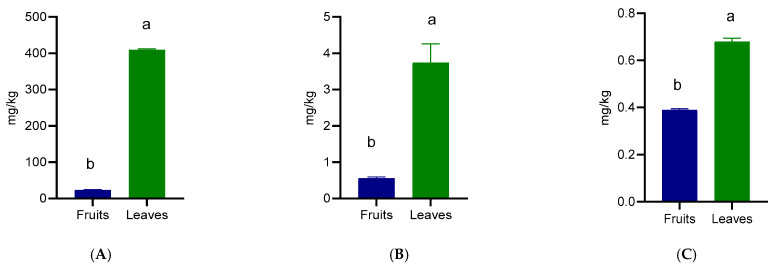
Xanthophyl content of wild *Prunus spinosa* stone fruits and leaves. Different letters (a,b) show significant differences at *p* < 0.05 according to ANOVA test; n = 3 represents 3 independent analyses, each performed with 3 analytical determinations. (**A**) Lutein, (**B**) Astaxanthin, (**C**) Canthaxanthin.

The carotenoid profile of *Prunus spinosa* fruits and leaves revealed significant differences in the concentrations of lutein, astaxanthin, and canthaxanthin, with leaves consistently presenting superior values compared to fruits (Figure 4). Lutein was the predominant carotenoid in both organs, reaching 409.38 mg/kg in leaves and 23.18 mg/kg in fruits (Figure 4A). Astaxanthin was also detected, with leaves containing 3.74 mg/kg compared to 0.56 mg/kg in fruits (Figure 4B). Canthaxanthin was detected at 0.68 mg/kg in leaves and 0.39 mg/kg in fruits (Figure 4C), showing a relatively low presence.

### 3.6. Tocopherol Profile of Wild Prunus Spinosa Stone Fruits and Leaves

The tocopherol profile of *Prunus spinosa* fruits and leaves is presented in Table 3. Significant differences (*p* < 0.0001) were observed between the two plant organs for all tocopherol isoforms. The α-tocopherol dominated both co-products. The fruits retained significantly lower γ and δ fractions, compared with leaves. However, the total vitamin E content in leaves was with 94.10% higher than in fruits.

### 3.7. Polyphenols Profile of Wild Prunus Spinosa Stone Fruits and Leaves

The polyphenols profile showed that the leaves are highly rich in phenolic acids (Table 4). The most abundant was chlorogenic acid, from the hydroxycinnamic class; while in the hydroxybenzoic acids no major compound was identified, important concentrations of hydroxybenzoic acid were identified in leaves. The leaves also contain significant amounts (*p* <0.05) of flavonoids, especially flavanols, which are powerful antioxidants found in plants generally. In the flavonols class, rutin was significantly (*p* <0.05) abundant in leaves. From the stilbene group, resveratrol was determined in higher concentrations in fruits but without significant effect.

## 4. Discussion

The higher CP content in the leaves can be explained by their physiological role as metabolically active organs, rich in chlorophyll-binding proteins, photosynthetic enzymes, and structural proteins. Similar findings have been reported in other species of the *Prunus* genus, such as *Prunus domestica,* where the leaves were reported to contain 13.10% CP, [24]. In contrast, fruits generally contain lower CP, since their primary function is reproductive and energetic storage rather than metabolic activity. Recently, Ozzengin et al. [8] reported 0.99% of CP for wild plum fruits, while *Prunus domestica* var. karaca and uryani fruits contained 0.87% and 1.26%. The EE values were relatively low in both organs, which is consistent with the general composition of *Prunus* fruits and leaves. Although the fruits contained slightly higher EE (4.11%) compared to leaves (3.07%), these results revealed higher CP than those reported previously in *Prunus spinosa* fruits (1.98%) from North-eastern Portugal [25]. These differences could be due to the lipid accumulation in fruits, which vary depending on species and environmental factors [17]. The CF was significantly higher in fruits (22.16%) than in leaves (13.86%). This difference reflects the structural role of dietary CF in stone fruit tissue, particularly in the pericarp and seed coats, which are rich in cellulose, hemicellulose, and lignin. Similar results have been reported in wild fruits, where CF values ranged between 3.86% and 19.50% [26]. In contrast, cultivated plums typically contain much lower dietary fiber, suggesting that wild relatives retain higher structural carbohydrate content. The lower CF content in leaves can be attributed to their softer tissues and the requirement for flexibility and photosynthetic efficiency, as opposed to the mechanical protection and dispersal functions of fruits. These findings align with the broader observation that wild fruits tend to provide higher CF and CP contents than cultivated varieties [27]. The complementary nutritional profiles of the two co-products of wild *Prunus spinosa* growing in Romania indicate their potential as nutritional resources for animal feeding and, with appropriate processing and evaluation, for human nutrition.

In terms of mineral content, the concentrations determined are within the range reported for seven Japanese *Prunus salicina* cultivars (2.9 to 11.2 mg/kg) [28], but lower than other *Prunus* species, such as *Prunus salicina* Lindl. and *Prunus domestica* L. leaves, as reported by Hamdani et al. [29]. The comparable copper levels in fruits and leaves suggest relatively uniform distribution, possibly reflecting the role of this micronutrient in redox enzymes and photosynthetic proteins. In contrast, manganese and iron were found at much higher concentrations in leaves than in fruits. The manganese content in leaves (43.57 mg/kg) was over six times higher than in fruits (7.02 mg/kg; *p* < 0.0001). Similarly, iron concentration was significantly higher in leaves (370.37 mg/kg) compared with fruits (44.87 mg/kg; *p* < 0.0001). Previous reports also highlight elevated manganese and iron levels in plum leaves compared with fruits [7], indicating that leafy tissues act as primary sinks for these elements due to their involvement in photosynthetic and oxidative metabolism. In human nutrition, manganese is an essential micronutrient required for bone development, regulation of carbohydrate and lipid metabolism, and as a cofactor in antioxidant enzymes such as manganese superoxide dismutase [30]. Although manganese deficiency is rare, the higher manganese levels observed in leaves highlight their nutritional relevance; however, the contribution of leafy tissues to manganese intake depends on their digestibility, bioavailability, and the presence of potential antinutritional factors. Iron on the other side is a critical element for hemoglobin and myoglobin synthesis, oxygen transport, and numerous redox enzymes, while its deficiency remains one of the most widespread nutritional disorders worldwide, leading to iron-deficiency anemia, fatigue, and impaired cognitive development [31]. In terms of iron intake, the measured concentrations suggest that both matrices could contribute to dietary iron intake (26%). However, iron bioavailability from plant-based sources is strongly influenced by the food matrix and the presence of absorption inhibitors. In this light, these co-products may represent a potential iron source for feed formulations and, following appropriate processing and safety assessment, for functional food applications. Zinc was also significantly higher in leaves (10.42 mg/kg) compared to fruits (8.54 mg/kg; *p* = 0.0212). Although the difference was less pronounced than for iron and manganese. Other authors found that the concentration of zinc in fruits ranged from 1.14 to 8.24 mg/kg [8], in *Prunus spinosa* and *Prunus domestica* cultivated in Turkey which is much lower compared with the Romanian wild *Prunus spinosa*. In leaves, the zinc concentration is in range with those reported by Hamdani et al. [29] in 27 plums varieties (2.55 to 25.18 mg/kg). These results are significant since regular zinc intake is crucial for growth and reproductive health, while deficiency can impair immunity and increase susceptibility to infections [31]. Therefore, fruits could represent a moderate dietary source of Zn.

The lipids profile in fruits obtained in the current study, are similar to those reported in plum kernel oils [32]. On *Prunus spinosa,* a recent work [5] reported similar concentrations of fatty acids in wild plum fruits, with a content of α-linolenic acid of 1.814 g/100 g, and slightly lower n-6/n-3 ratio (15.56). Wild plums kernel oils reported linoleic of 57% and oleic of 18% as the two major fatty acids [15]. The higher level of α-linolenic acid is attributed to the chloroplast-rich tissues where trienoic fatty acids maintain membrane fluidity and enable photoprotective and oxidative-stress responses. The literature data showed that α-linolenic acid is the dominant fatty acid in leaves, and increase under different stress factors (light, cold, drought) [33,34]. Compared with traditional oilseeds (i.e., flaxseed, chia, and rapeseed), commonly used in designing rich fat diets [9], the fatty profile of leaves is exceptional due to the high α-linolenic acid content, which typically comprise both oleic and linoleic acids [17,35]. In terms of the lipid profile of *Prunus* species, reports on leaves are scarcer than those reporting kernel oil reports; however, in a comparative study between fruit, leaves, and pomace of black chokeberry, Saracila et al. [11] found that the fruits are richer in linoleic and oleic acids, while the leaves contain a high content of α-linolenic acid (29%). For other berry leaves, Varzaru et al. [13] reported that blackberry and raspberry leaves are likewise rich in α-linolenic acid, although the individual contents vary by species and environment. These results are also in line with those observed in wild *Prunus spinosa* fruits and leaves from the current study. The predominance of essential α-linolenic acid in leaves is explained by the presence of chloroplast galacto- and phospholipid requirements for thylakoid fluidity and photosynthetic function [36]. On the other hand, fruit triacylglycerols in many drupes, berries, and fruits accumulate more oleic and linoleic acids, favoring energy storage and softer matrices. These differences are clearly highlighted in different comparative studies; however, they are less explained. From a consumer health perspective, these differences are of significant importance. The fruit oil profile, containing higher MUFA with substantial linoleic content, supports cardiometabolic benefits linked to oleic (improved lipid profile and oxidative stability of the matrix) while maintaining a conventional n-6/n-3 ratio typical of many edible fruits. The leaves’ lipid pattern, with higher α-linolenic acid and PUFAs, and a low n-6/n-3, is desirable for inflammation resolution and cardiovascular protection. Potentially used as dried powders or extracts, leaves could complement plant-based products by lowering dietary n-6/n-3. However, potential applications of leaf-derived lipids, such as in dried powders or extracts, would require careful evaluation of safety, oxidative stability, and lipid bioaccessibility before consideration in food formulations.

In a recent study, Veličković et al. [37] highlighted the significant total polyphenol content (181.19 mg GAE) in *Prunus spinosa* leaves and also a higher DPPH radical scavenging activity than fruits, which is in line with current findings. Other reports, also showed that *Prunus spinosa* fruits are richer in anthocyanins and ascorbic acid, giving them a notable antioxidant activity in DPPH/ABTS tests and an important nutra-functional potential, but these values depend on the extraction method, ripening stage of the fruits and leaves, as well as extraction methods [38,39]. The leaves, due to their high polyphenol content and DPPH scavenging activity, represent a promising source of antioxidant compounds; however, their application as natural ingredients in the food industry requires careful evaluation. These compounds could be used as additives in functional beverages, or for improving the stability in of oils and bakery products, offering alternatives to synthetic antioxidants. In the case of leaf-derived extracts, particular attention should be given to safety assessments, as high polyphenol concentrations may exert pro-oxidant effects or interfere with nutrient absorption at elevated doses. Therefore, further studies addressing toxicity thresholds, bioaccessibility, and regulatory aspects are required before considering their use in food or nutraceutical applications.

The high lutein concentration in leaves is consistent with their photosynthetic activity, since lutein plays a central role in photoprotection by dissipating excess light energy and preventing oxidative damage in chloroplasts. In comparison with other leafy sources, such as spinach or kale, where lutein levels typically range between 60 and 120 mg/kg, the values recorded in *Prunus spinosa* leaves are several-fold higher, highlighting their potential as an extraordinary natural source of this xanthophyll [40,41]. From a nutritional perspective, lutein is a well-characterized dietary carotenoid involved in visual function, particularly through its accumulation in the macula, where it contributes to blue-light filtering and antioxidant protection. Clinical studies suggest that intakes as low as 6 mg/day can exert protective effects, and therefore the high concentrations observed in wild plum leaves reveal their potential use in nutraceuticals or functional food formulations [42]. However, translating leaf lutein concentrations into effective dietary exposure depends on processing, bioavailability, and safety considerations. Astaxanthin was also detected, with leaves containing 3.74 mg/kg compared to 0.56 mg/kg in fruits (Figure 4b). In blackberry and raspberry leaves, astaxanthin was more abundant in the first one (38.52 mg/kg), compared with raspberry (7.441 mg/kg), as reported by others [13]. However, the black chokeberry leaves contain 100.29 mg/kg [11], which contradicts the mention of Oslan et al. [43], which reported that astaxanthin presence in terrestrial plants is unusual. The detection of this carotenoid is noteworthy, as astaxanthin is widely recognized for its strong antioxidant capacity in various biological systems [44]; however, its physiological relevance when derived from terrestrial plant matrices remains insufficiently characterized. Canthaxanthin was detected at 0.68 mg/kg in leaves and 0.39 mg/kg in fruits, showing a relatively low presence, but its contribution to the overall carotenoid portfolio adds further antioxidant diversity, especially since this compound is produced mainly by certain algae, fungi, and bacteria with limited occurrence in higher plants. However, the low amounts of this keto-carotenoid observed may contribute to the overall carotenoid profile without posing a risk of excessive intake, potentially acting synergistically with tocopherols and phenolic compounds [45]. Studies have shown that this bioactive compound is not evenly present in the same plant species. For example, black elderberry leaves from the northern Romanian part contain 1.244 mg/kg while the southern leaves contain up to 162.54 mg/kg, showing a very high variability [17]. While data on these compounds in *Prunus* species are almost absent, these results add novelty, revealing the potential usage of these co-products. Nevertheless, further studies are required to assess bioavailability, safety, and appropriate processing before considering any nutritional application.

The α-tocopherol dominated both organs, especially because α is the major leafy tocopherol, whereas γ predominates in many seeds and seed oils [46]. The fruits retained measurable γ and δ fractions (25–35% of total), similar to distributions observed in tomato fruits and *Prunus* seed oils [4,47]. Leaves contained significantly higher (*p* < 0.05) levels of the α-tocopherol isoform (1214.98 mg/kg DW) compared to fruits (49.97 mg/kg), being the most biologically active form of vitamin E in humans and playing a critical role in maintaining membrane integrity by protecting polyunsaturated fatty acids from peroxidation [47]. The high α-tocopherol concentration in leaves is consistent with their need for enhanced antioxidant protection against photooxidative stress during photosynthesis [48]. Both δ- and γ-tocopherol concentrations were significantly higher in leaves than in fruits. These isoforms also contribute to the antioxidant potential, with δ-tocopherol being particularly effective in scavenging reactive nitrogen species, while γ-tocopherol is known for its synergistic antioxidant role in combination with α-tocopherol isoform [45]. The total tocopherol content in leaves (1268.24 mg/kg DW) was more than 16-fold higher than in fruits (74.75 mg/kg DW), which highlight their adaptive mechanism for stress resistance and survival under fluctuating environmental conditions [48]. In other leaves, such as blackberry or raspberry, Varzaru et al. [13] reported a content of 179.9 mg/kg and 149.7 mg/kg, respectively, while in black chokeberry leaves the content reported by Saracila et al. [11] was 1172.20 mg/kg, which is closer to the content determined in *Prunus spinosa*. These differentiated results indicate that plants that grow in harsher environments utilize more in photoprotection and antioxidants, leading to higher vitamin E deposition, compared with those that are shade-adapted and rely less on vitamin E for photoprotection. Fruits, although containing lower levels, still provide nutritionally valuable tocopherols. These co-products provide more than the European Food Safety Authority (EFSA) recommendations for daily dietary references (13 mg α-tocopherol/day for men and 11 mg/day for women) [49]. The significant tocopherol content in *Prunus spinosa* leaves suggests potential applications as a source of natural antioxidants in nutraceutical formulations or functional foods. While consumption of leaves is less common in the human diet, their extractable compounds may be of interest for supplement development.

Regarding the polyphenol profile of *Prunus spinosa*, it was noted that leaves contain some classes of polyphenols in concentrations similar to or even higher than those in fruits. Similarly to our study, Veličković et al. [37] reported the identified phenolic profile consisting of phenolic acids and flavonoids. Although most of the works focus on fruits, it was revealed that the phenolic profile is dominated by hydroxycinnamic acids, flavonoids (flavonols), anthocyanins, proanthocyanidins, and, in some cases, flavan-3-ols [50,51]. Magiera et al. [39] showed that out of 57 phenolic compounds identified in *Prunus spinosa* fruits, the phenolic acids (up to 92 mg/g), flavonoids (up to 41 mg/g), condensed proanthocyanidins, and anthocyanins (up to 9.2 mg/g) were the main contributors to the total polyphenol content. However, it is important to emphasize that in most works the detailed phenolic profile targets the fruits, while the data for leaves are often limited to total content or spectrophotometric antioxidant assays.

Similar observations highlighting the scarcity and inconsistency of the available literature data, particularly for leaves, have also been reported by others [17,37,51]. Therefore, further research is required to comprehensively characterize the phenolic composition of these co-products, particularly leaves, using targeted and non-targeted analytical approaches. In addition, studies addressing phenolic bioaccessibility, potential antinutritional interactions, and dose-dependent effects are necessary to better understand their physiological relevance. Such investigations would support a more accurate assessment of both the benefits and limitations associated with leaf-derived phenolic compounds of wild *Prunus spinosa* co-products.

## 5. Conclusions and Limitations

*Prunus spinosa* stone fruits and leaves show complementary nutritional profiles. The fruits are richer in crude protein, crude fiber, monounsaturated fatty acids, and iron. The leaves contain significantly higher levels of tocopherols, lutein, α-linolenic acid, and trace minerals, especially manganese, zinc, and iron. Both fruits and leaves can serve as valuable sources of natural antioxidants and functional lipids, supporting their potential use in functional foods and nutraceuticals.

Beyond their traditional use in liqueur production, the results of the present study suggest that *Prunus spinosa* L. fruits may have potential as food ingredients or nutraceutical raw materials. The fruits could be consumed fresh or processed (e.g., as juices, jams, or dried products), while from a nutraceutical perspective, the use of standardized extracts may be particularly suitable to ensure a controlled intake of biologically active compounds.

However, while the leaves may be used in dried form for herbal infusions, the direct use of leaves as food ingredients may be limited by sensory characteristics such as bitterness or astringency, as well as by the presence of potentially unfavorable compounds, which were not evaluated in the present study. Therefore, further investigations addressing antinutritional factors, the bioavailability of compounds, sensory acceptability, and safety, are necessary before broader food applications can be recommended.

## Figures and Tables

**Table 1:** Minerals composition of wild *Prunus spinosa* stone fruits and leaves.

Items	Fruits	Leaves	SEM	*p* Value
mg/kg				
Copper	3.47 a	3.28 a	0.055	0.0951
Manganese	7.02 b	43.57 a	0.122	0.0001
Iron	44.87 b	370.37 a	0.033	0.0001
Zinc	8.54 b	10.42 a	0.101	0.0212

Different letters (a,b) show significant differences at *p* < 0.05 according to ANOVA test; SEM represents the standard error of the mean calculated from the comparative analysis between fruits and leaves; n = 3 represents 3 independent analyses, each performed with 3 analytical determinations.

**Table 2:** Fatty acid composition of wild *Prunus spinosa* stone fruits and leaves.

Fatty Acids	Carbon Bond	Fruits	Leaves	SEM	*p* Value
g/100 g FAME					
Caprylic	C 8:0	0.18 b	0.72 a	0.378	0.0001
Capric	C 10:0	0.16 b	0.76 a	0.428	0.0001
Lauric	C 12:0	nd	1.08	-	-
Myristic	C 14:0	0.22 b	0.98 a	0.534	0.0022
Palmitic	C 16:0	7.56 b	28.2 a	14.56	0.0001
Palmitoleic	C 16:1	0.92 b	1.35 a	0.308	0.0105
Heptadecenoic	C 17:1	0.12	nd	-	-
Stearic	C 18:0	1.92 b	7.72 a	4.105	0.0001
Oleic cis	C 18:1	55.8 a	15.6 b	28.45	0.0001
Linoleic cis	C 18:2n6	30.3 a	18.5 b	8.39	0.0001
Linolenic γ	C 18:3n6	0.04	nd	-	-
Linolenic α	C 18:3n3	1.61 b	25.2 a	16.71	0.0001
Octadecatetraenoic	C18:4n3	0.17	nd	-	-
Eicosadienoic	C 20:2n6	0.33	nd	-	-
SFA		10.01 b	38.33 a	8.501	0.0001
MUFA		56.83 a	16.92 b	18.22	0.0001
PUFA		32.52 b	43.74 a	7.944	0.0204
UFA		89.36 a	60.66 b	16.28	0.0001
SFA/UFA		0.12 b	0.62 a	0.368	0.0001
PUFA/MUFA		0.61 b	2.61 a	1.425	0.0001
n-3		1.83 b	25.2 a	10.59	0.0001
n-6		30.73 a	18.5 b	8.648	0.0001
n-6/n-3		17.30 a	0.74 b	11.70	0.0001

SFA—saturated fatty acids; MUFA—monounsaturated fatty acids; PUFA—polyunsaturated fatty acids; UFA—unsaturated fatty acids; different letters (a,b) in the same row show significant differences at *p* < 0.05 according to ANOVA test; SEM represents the standard error of the mean calculated from the comparative analysis between fruits and leaves.; n = 3 represents 3 independent analyses, each performed with 3 analytical determinations.

**Table 3:** Tocopherols content of wild *Prunus spinosa* stone fruits and leaves.

Items	Fruits	Leaves	SEM	*p* Value
mg/kg				
α-tocopherol	49.97 b	1214.98 a	6.025	0.0001
δ-tocopherol	6.01 b	13.61 a	1.493	0.0001
γ-tocopherol	18.76 b	39.65 a	2.173	0.0001
Σ tocopherols	74.75 b	1268.24 a	9.554	0.0001

Different letters (a,b) show significant differences at *p* < 0.05 according to ANOVA test; SEM represents the standard error of the mean calculated from the comparative analysis between fruits and leaves.; n = 3 represents 3 independent analyses, each performed with 3 analytical determinations.

**Table 4:** Polyphenols profile of wild *Prunus spinosa* stone fruits and leaves.

Items	Fruits	Leaves	SEM	*p* Value
mg/g				
**Σ Phenolic Acids**	**0.633 b**	**24.498 a**	0.053	0.0001
**Σ *Hydroxybenzoic acids***	**0.303 b**	**0.813 a**	0.051	0.0212
Gallic acid	0.083 a	0.052 b	0.044	0.0058
Vanillic acid	0.005 b	0.032 a	0.023	0.0001
Syringic acid	0.007 b	0.176 a	0.099	0.0001
Hydroxybenzoic acid	0.187 b	0.384 a	0.140	0.0001
Ellagic acid	0.013 b	0.167 a	0.156	0.0001
Protocatechuic acid	0.009 a	0.003 a	0.022	0.0513
**Σ *Hydroxycinnamic acids***	**0.330 b**	**23.685 a**	0.341	0.0001
Chlorogenic acid	0.274 b	23.502 a	0.362	0.0001
Caffeic acid	0.010 b	0.028 a	0.045	0.0109
Methoxycinnamic acid	0.002 b	0.014 a	0.055	0.0327
Ferulic acid	0.025 b	0.089 a	0.084	0.0001
Coumaric acid	0.015 b	0.048 a	0.093	0.0001
Cinnamic acid	0.004 a	0.003 a	0.010	0.1002
**Σ Flavonoids**	**0.376 b**	**6.082 a**	0.259	0.0001
***Σ Flavanols***	***0.292 b***	***1.983 a***	0.264	0.0001
Epigallocatechin	0.044 b	0.662 a	0.093	0.0001
Catechin	0.093 b	0.643 a	0.075	0.0001
Epicatechin	0.155 b	0.679 a	0.020	0.0001
***Σ Flavonols***	***0.068 b***	***4.019 a***	0.197	0.0001
Rutin	0.066 b	3.992 a	0.186	0.0001
Quercetin	0.001 b	0.017 a	0.059	0.0001
Kaempferol	0.002 a	0.010 a	0.044	0.0503
***Σ Anthocyanins***	***0.016 b***	***0.079 a***	0.069	0.0001
Cyanidine-3-glucoside	0.016 b	0.079 a	0.069	0.0001
**Σ Stilbene**	**0.007 a**	**0.005 a**	0.010	0.0605
Resveratrol	0.007 a	0.005 a	0.010	0.0605

Different letters (a,b) show significant differences at *p* < 0.05 according to ANOVA test; SEM represents the standard error of the mean calculated from the comparative analysis between fruits and leaves.; n = 3 represents 3 independent analyses, each performed with 3 analytical determinations.

## Data Availability

The original contributions presented in the study are included in the article, further inquiries can be directed to the corresponding author.
